# Lack of Susceptibility to SARS-CoV-2 and MERS-CoV in Poultry 

**DOI:** 10.3201/eid2612.202989

**Published:** 2020-12

**Authors:** David L. Suarez, Mary J. Pantin-Jackwood, David E. Swayne, Scott A. Lee, Suzanne M. DeBlois, Erica Spackman

**Affiliations:** US Department of Agriculture, Athens, Georgia, USA

**Keywords:** coronavirus disease, SARS-CoV-2, severe acute respiratory syndrome coronavirus 2, severe acute respiratory syndrome, SARS, viruses, respiratory infections, zoonoses, COVID-19, SARS-related coronavirus, Middle East respiratory syndrome coronavirus, MERS-CoV, poultry, mammalian beta-coronavirus, poultry health, chicken, turkey, goose, quail, duck

## Abstract

We challenged chickens, turkeys, ducks, quail, and geese with severe acute respiratory syndrome coronavirus 2 or Middle East respiratory syndrome coronavirus. We observed no disease and detected no virus replication and no serum antibodies. We concluded that poultry are unlikely to serve a role in maintenance of either virus.

Coronaviruses of animals periodically transmit to humans (*1*), as recently occurred with severe acute respiratory syndrome coronavirus 2 (SARS-CoV-2). SARS-CoV-2 was recognized in December 2019 in cases of atypical pneumonia in hospitalized patients in Wuhan, China. The virus is a novel betacoronavirus, related to the now-eradicated severe acute respiratory syndrome coronavirus (SARS-CoV) from 2003, with which SARS-CoV-2 has 82% identity across the genome (*2*). SARS-CoV-2 is highly transmissible among humans and particularly virulent for elderly persons and those with certain underlying health conditions. Multiple studies have examined the susceptibility of domestic animals to SARS-CoV-2 to establish the risk for zoonotic transmission; 2 studies have shown chickens and Pekin ducks were not susceptible to infection (*3**,**4*).

Middle East respiratory syndrome coronavirus (MERS-CoV), another coronavirus of high concern associated with zoonotic infection, was first detected in patients with severe acute lower respiratory tract disease in Saudi Arabia in 2012. MERS-CoV causes lower respiratory disease, similar to the SARS-CoVs (*5*). Unlike SARS-CoV-2, MERS-CoV transmits poorly to humans and does not exhibit sustained human-to-human transmission; however, it has a high case fatality rate of »30%. Although the MERS-CoV case count is low, human cases continue to be reported, therefore there is a possibility for the virus to adapt to humans.

Based on sequence similarity, the closest relatives of SARS-CoV-2 and MERS-CoV are believed to be bat betacoronaviruses (*6*); the sequence difference between human and bat isolates suggests the existence of an intermediary host. For MERS-CoV, dromedary camels appear to be the primary natural reservoir of infection to humans, but other domestic animals seem to be susceptible to infection (*7*,*8*). Hemida et al. looked for MERS-CoV antibodies in chickens; all samples were negative (*9*).

Because poultry are so widespread and have close and extended contact with humans and other mammals in many production systems, including live animal markets, we conducted susceptibility studies with SARS-CoV-2 and MERS-CoV in 5 common poultry species. Embryonating chicken eggs (ECE) have been used for virus isolation culture, including use in vaccine production, for diverse avian and mammalian viruses; therefore, we tested ECE for their ability to support the replication of both viruses.

We examined 5 poultry species: chickens (*Gallus gallus domesticus*), turkeys (*Meleagris gallopavo*), Pekin ducks (*Anas platyrhinchos domesticus*), Japanese quail (*Coturnix japonica*), and white Chinese geese (*Anser cygnoides*). The US National Poultry Research Center Institutional Animal Care and Use Committee reviewed and approved all procedures involving animals; the Institutional Biosafety Committee approved the use of the viruses.

To evaluate their susceptibility to these viruses, 10 birds of each species were challenged with a virus isolate obtained from the Biodefense and Emerging Infections Research Resources Repository (BEI Resources; National Institute of Allergy and Infectious Diseases, National Institutes of Health). We used either the USA-WA1/2020 isolate of SARS-CoV-2 (BEI NR-58221) or the Florida/USA-2_SaudiArabia_2014 isolate of MERS-CoV (BEI NR-50415) (Appendix). 

We collected oropharyngeal and cloacal swabs from all birds at 2, 4, and 7 days postchallenge (dpc) and tested them for virus by real-time reverse transcription PCR. At 14 dpc we collected serum specimens from the birds and tested for antibody to the challenge virus by microneutralization. No clinical signs were observed at any time in any species, and virus was not detected in any swab material (Table). Antibodies were not detected in serum from any birds at 14 dpc. These results suggest that neither virus replicated in any of the avian species evaluated or that they replicated at a level that was too low to be detected.

**Table:** Poultry testing positive for SARS-CoV-2 or MERS-CoV, United States*

Species	SARS-COV-2										MERS-CoV								
	No. positive at 2 dpc			No. positive at 4 dpc			No. positive at 7 dpc				No. positive at 2 dpc			No. positive at 4 dpc			No. positive at 7 dpc		
	OP	CL		OP	CL		OP	CL	Antibody		OP	CL		OP	CL		OP	CL	Antibody
Chickens (*Gallus gallus domesticus*)	0	0		0	0		0	0	0		0	0		0	0		0	0	0
Turkeys (*Meleagris gallopavo*)	0	0		0	0		0	0	0		0	0		0	0		0	0	0
Japanese quail (*Coturnix japonica*)	0	0		0	0		0	0	0		0	0		0	0		0	0	0
Pekin ducks (*Anas platyrhinchos*)	0	0		0	0		0	0	0		0	0		0	0		0	0	0
Chinese domestic geese (*Anser cygnoides*)	0	0		0	0		0	0	0		0	0		0	0		0	0	0

*Real-time reverse transcription PCR was used to test the oropharyngeal and cloacal swabs collected from 10 individuals of each poultry species inoculated with SARS-CoV-2 or MERS-CoV. We tested serum samples for antibody 14 dpc by virus neutralization assay. Three birds of each species served as noninoculated controls. CL, cloacal swab; dpc, days postchallenge; MERS-CoV, Middle East respiratory syndrome coronavirus; OP, oropharyngeal swab; SARS-CoV-2, severe acute respiratory syndrome coronavirus 2.

We tested ECE for their ability to support SARS-CoV-2 or MERS-CoV replication after inoculation with any of the 3 most common routes: yolk sac, chorioallantoic sac, or chorioallantoic membrane (Appendix). We collected yolk, allantoic fluid (albumin), and embryo tissues from inoculated eggs; we tested for viral replication by attempting virus isolation in Vero cells from the egg material after each of 2 ECE passages. We did not recover either virus in Vero cells from the inoculated ECEs, nor did we observe lesions in any of the embryos inoculated with SARS-CoV-2 or MERS-CoV. The ECE results with SARS-CoV-2 are consistent with the results reported by Barr et al. (*10*).

Identifying potential reservoir hosts of the novel coronaviruses is critical to controlling exposure and subsequent infection, as well as to preserving a safe and consistent food supply. None of the avian species nor the ECE appeared to support replication of either virus. Our findings demonstrate that poultry are unlikely to serve a role in the maintenance or transmission of either SARS-CoV-2 or MERS-CoV, and furthermore that ECE are not a viable laboratory host system.

Appendix Additional information about susceptibility of poultry to SARS-CoV-2 and MERS-CoV.
