# Supplementary material for: Lack of Susceptibility to SARS-CoV-2 and MERS-CoV in Poultry
Source: Emerg Infect Dis. 2020 Dec;26(12):3074–6. doi: 10.3201/eid2612.202989 (PMC7706925; doi:10.3201/eid2612.202989)
Supplement: Appendix — Additional information about susceptibility of poultry to SARS-CoV-2 and MERS-CoV. [file 20-2989-Techapp-s1.pdf]

Article DOI: https://doi.org/10.3201/eid2612.202989

Lack of Susceptibility to SARS-CoV-2 and MERS-CoV in Poultry

Appendix

Detailed Methods

Viruses

The USA-WA1/2020 (BEI NR-58221) (*1*) isolate of severe acute respiratory syndrome coronavirus 2 (SARS-CoV-2) and the Florida/USA-2_SaudiArabia_2014 isolate of Middle East respiratory syndrome coronavirus (MERS-CoV) (BEI NR-50415) (*2*) were both obtained from Biodefense and Emerging Infections Research Resources Repository, National Institute of Allergy and Infectious Diseases, National Institutes of Health. Both viruses were propagated and titrated in CCL-81 Vero cells (International Reagent Resource FR-243). SARS-CoV-2 was utilized at 5 total passages in Vero cells, and MERS-CoV was utilized at 6 total passages in Vero cells. Viruses were used under the approval of the US National Poultry Research Center Institutional Biosafety Committee.

Evaluation of Virus Replication in Avian Species

Five poultry species were selected because of their prevalence worldwide: chickens (*Gallus gallus domesticus*), turkeys (*Meleagris gallopavo*), Pekin ducks (*Anas platyrhinchos domesticus*), Japanese quail (*Coturnix japonica*), and in wet markets in China: chickens, Pekin ducks, quail, and Chinese domestic geese (*Anser cygnoides*). Chickens and turkeys were obtained from in-house specific pathogen free (SPF) flocks. Ducks, geese, and quail were obtained from a commercial hatchery. The US National Poultry Research Center Institutional Animal Care and Use Committee reviewed and approved all procedures involving animals.

The experimental design was informed by prior work with testing poultry for the susceptibility and pathogenesis of other novel viruses, avian coronaviruses, or viruses with similar expected pathogenesis (i.e. respiratory tract infection here) in poultry (*3-8*). We aimed to use a high dose; for most viruses 10^{5}–10^{6} infectious units is adequate to achieve infection and is

generally not too artificially high for the dose to which an animal would be exposed to in the real world. The simulated respiratory route was utilized, which would mimic a natural route of infection.

Each bird was individually tagged for identification. For each species 10 birds were challenged with each virus and 3 birds were not inoculated to serve as age-matched controls.

Blood was collected from all birds immediately prior to infection and was tested by microneutralization for antibodies to the appropriate challenge virus. Chickens, turkeys, and quail were challenged at 4 weeks of age; ducks and geese were challenged at 2 weeks of age (Appendix Table). Chickens, turkeys, and quail were challenged with 5.4 log_{10} 50% tissue culture infectious doses ($TCID_{50}$) of SARS-CoV-2 in 0.1mL or 5.2log_{10} $TCID_{50}$ of MERS-CoV in 0.1mL by the intrachoanal route. Ducks and geese were challenged with 6.0 log_{10} $TCID_{50}$ of SARS-CoV-2 or 5.5 log_{10} $TCID_{50}$ of MERS-CoV, each in 0.1mL by the intrachoanal route. Birds were observed a minimum of daily for clinical signs.

Oropharyngeal (OP) and cloacal (CL) swabs were collected from all challenged birds at 2, 4, and 7 days post challenge (DPC) and were tested for virus by real-time reverse transcription RT-PCR. The rRT-PCR was run with the 2, 4, and 7 DPC samples immediately after the 7 DPC samples were collected. Because they were negative, we determined that it was not necessary to test at any later time points.

Because there was no evidence of infection and no clinical signs, and no virus was excreted by the respiratory tract or intestinal tracts, lesions were not expected to have developed, therefore no birds were necropsied during the course of the study.

At 14 DPC blood was collected from all surviving birds and the serum samples were tested by microneutralization to evaluate whether there was an antibody response to the challenge virus.

Replication in Embryonating Chicken's Eggs

Embryonating chicken eggs (ECE) were evaluated for their ability to support replication of SARS-CoV-2 and MERS-CoV. Procedures were identical for both viruses. Five ECE were inoculated with $10^{6.5}$ $TCID_{50}$ in 0.2mL for each of the 3 most common routes of inoculation:

yolk sac (YS), chorioallantoic sac (CAS), and chorioallantoic membrane (CAM). Established inoculation procedures for each route were utilized (*9*). Eggs were candled daily for viability.

Samples were collected from the inoculated eggs when the embryo was found to be nonviable or at the end of the incubation period. Yolk, allantoic fluid/albumin, embryo tissue (2–3 grams of viscera and thigh muscle) were collected from YS inoculated eggs. Allantoic fluid/albumin, embryo tissues were collected from CAS inoculated eggs, and allantoic fluid/albumin, embryo tissues, and egg membrane were collected from CAM inoculated eggs. During sample collection the embryos were dissected to observe lesions. Age-matched noninoculated ECE served as controls.

CAM and embryo tissues were homogenized in PBS with glass beads in a FastPrep 24 (MP Biomedical LLC, https://www.mpbio.com) then was centrifuged at 17 Kxg for 10 minutes and the supernatant was used for the second passage and for RNA extraction for subsequent testing by rRT-PCR. Allantoic fluid/albumin was used directly for the second passage and RNA extraction. To complete the second passage, all sample material from the 5 eggs of same inoculation route were pooled. The material was then inoculated identically to the first passage. Material from both passages was tested by inoculation into Vero cells in triplicate for fluid and embryo material from each inoculation route as described above to test for the presence of virus.

Microneutralization

Virus microneutralization with serum from each species was conducted with both SARS-CoV-2 and MERS-CoV in CCL-81 Vero cells as described by Algaissi and Hashem (*10*), with the modifications that the dilutions of serum tested were 1:4, 1:8, 1:16, and 1:32 and that the antibody-treated virus was added when the cells were plated. Titers >1:8 were considered positive. Positive control antibodies were commercially available monoclonal antibodies to the S2 region of the spike protein: SARS-CoV-2 used at 12.5µg/mL (MP Biomedical), and MERS-CoV used at 20µg/mL (EastCoast Bio, https://eastcoastbio.com).

RNA Extraction and Quantitative Real-Time RT-PCR

RNA was extracted from OP and CL swab material with the Ambion Magmax kit (ThermoFisher, https://thermofisher.com) as described previously (*11*). The rRT-PCR primers, probe, and cycling conditions for SARS-CoV-2 for the N1 primer and probe set from the US Centers for Disease Control were utilized (*12*). The N3 primers, probe, and conditions reported

by Lu et al. which target the nucleoprotein gene was used for MERS-CoV detection (*13*). The AgPath ID one-step RT-PCR kit was used and the RT step of the reaction conditions was modified to accommodate the recommended kit conditions (PCR conditions recommended for each primer and probe set from the original protocols were used). A standard curve of RNA titrated virus was run in duplicate with each run of rRT-PCR to estimate titer equivalents of virus present in samples.

References

1. Gralinski LE, Menachery VD. Return of the coronavirus: 2019-nCoV. Viruses. 2020;12:135. PubMed https://doi.org/10.3390/v12020135
2. Bialek SR, Allen D, Alvarado-Ramy F, Arthur R, Balajee A, Bell D, et al.; Centers for Disease Control and Prevention (CDC). First confirmed cases of Middle East respiratory syndrome coronavirus (MERS-CoV) infection in the United States, updated information on the epidemiology of MERS-CoV infection, and guidance for the public, clinicians, and public health authorities - May 2014. MMWR Morb Mortal Wkly Rep. 2014;63:431–6. PubMed
3. Swayne DE, Beck JR, Zaki S. Pathogenicity of West Nile virus for turkeys. Avian Dis. 2000;44:932–7. PubMed https://doi.org/10.2307/1593067
4. McKinley ET, Spackman E, Pantin-Jackwood MJ. The pathogenesis of H3N8 canine influenza virus in chickens, turkeys, and ducks. Influenza Other Respir Viruses. 2010;4:353–6. PubMed https://doi.org/10.1111/j.1750-2659.2010.00165.x
5. Swayne DE, Suarez DL, Spackman E, Tumpey TM, Beck JR, Erdman D, et al. Domestic poultry and SARS coronavirus, southern China. Emerg Infect Dis. 2004;10:914–6. PubMed https://doi.org/10.3201/eid1005.030827
6. DeJesus E, Costa-Hurtado M, Smith D, Lee DH, Spackman E, Kapczynski DR, et al. Changes in adaptation of H5N2 highly pathogenic avian influenza H5 clade 2.3.4.4 viruses in chickens and mallards. Virology. 2016;499:52–64. PubMed https://doi.org/10.1016/j.virol.2016.08.036
7. Spackman E, Pantin-Jackwood M, Swayne DE, Suarez DL, Kapczynski DR. Impact of route of exposure and challenge dose on the pathogenesis of H7N9 low pathogenicity avian influenza virus in chickens. Virology. 2015;477:72–81. PubMed https://doi.org/10.1016/j.virol.2015.01.013

8. Brown JD, Stallknecht DE, Valeika S, Swayne DE. Susceptibility of wood ducks to H5N1 highly pathogenic avian influenza virus. J Wildl Dis. 2007;43:660–7. PubMed https://doi.org/10.7589/0090-3558-43.4.660

9. Spackman E, Stephens C. Virus isolation and propagation in embryonated eggs. In: Williams SM, editor. A laboratory manual for the isolation, identification and characterization of avian pathogens. 6th ed. Jacksonville (FL): American Association of Avian Pathologists; 2016. p. 361–8.

10. Algaissi A, Hashem AM. Evaluation of MERS-CoV neutralizing antibodies in sera using live virus microneutralization assay. Methods Mol Biol. 2020;2099:107–16. PubMed https://doi.org/10.1007/978-1-0716-0211-9_9

11. Das A, Spackman E, Pantin-Jackwood MJ, Suarez DL. Removal of real-time reverse transcription polymerase chain reaction (RT-PCR) inhibitors associated with cloacal swab samples and tissues for improved diagnosis of avian influenza virus by RT-PCR. J Vet Diagn Invest. 2009;21:771–8. PubMed https://doi.org/10.1177/104063870902100603

12. Lu X, Wang L, Sakthivel SK, Whitaker B, Murray J, Kamili S, et al. US CDC real-time reverse transcription PCR panel for detection of severe acute respiratory syndrome coronavirus 2. Emerg Infect Dis. 2020;26:1654–65. PubMed https://doi.org/10.3201/eid2608.201246

13. Lu X, Whitaker B, Sakthivel SK, Kamili S, Rose LE, Lowe L, et al. Real-time reverse transcription-PCR assay panel for Middle East respiratory syndrome coronavirus. J Clin Microbiol. 2014;52:67–75. PubMed https://doi.org/10.1128/JCM.02533-13

Appendix Table. Age at challenge and dose for each virus by species in study of SARS-CoV-2 and MERS-CoV in poultry

Species	Age at challenge, wk	Titer of challenge with SARS-CoV-2 (log_{10} $TCID_{50}$/bird)	Titer of challenge with MERS-CoV (log_{10} $TCID_{50}$/bird)
Chickens (*Gallus gallus domesticus*)	4	5.4	5.2
Turkeys (*Meleagris gallopavo*)	4	5.4	5.2
Japanese quail (*Coturnix japonica*)	4	5.4	5.2
Pekin ducks (*Anas platyrhinchos)*	2	6.0	5.5
Chinese domestic geese (*Anser cygnoides*)	2	6.0	5.5

*(TCID50 = 50% tissue culture infectious dose). MERS-CoV Middle East respiratory syndrome coronavirus; SARS-CoV-2, severe acute respiratory syndrome coronavirus 2.
